# Smoking and attitudes towards its cessation among native and international dental students in Lithuania

**DOI:** 10.1186/s12903-017-0397-y

**Published:** 2017-07-11

**Authors:** Apolinaras Zaborskis, Aiste Volkyte, Julija Narbutaite, Jorma I. Virtanen

**Affiliations:** 1 Institute of Health Research, Faculty of Public Health, Lithuanian University of Health Sciences, Tilzes 18, LT-47181 Kaunas, Lithuania; 2 Faculty of Odontology, Lithuanian University of Health Sciences, Luksos-Daumanto 6, LT-50106 Kaunas, Lithuania; 3 Lithuanian University of Health Sciences Hospital, Kaunas, Lithuania; 4 Research Unit of Oral Health Sciences, Faculty of Medicine, University of Oulu, P.O. Box 5000, FI-90014 Oulu, Finland; 5 Medical Research Center, Oulu University Hospital, Oulu, Finland

**Keywords:** Dental students, Smoking, Attitude of health personnel, Culture

## Abstract

**Background:**

Dental professionals are uniquely positioned to discourage smoking among their patients. However, little is known about the role of cultural background and attitudes towards smoking in the education of these professionals. Our study aimed to compare native Lithuanian and international dental students’ smoking habits, knowledge about the harmfulness of smoking and attitudes towards smoking cessation.

**Methods:**

We conducted a cross-sectional survey of smoking and its cessation among dental students at the Lithuanian University of Health Sciences (Kaunas, Lithuania) in 2012. All Lithuanian and international dental students in each year of dental school were invited to participate in the survey during a compulsory practical class or seminar. Altogether 606 students participated in the survey with a response rate of 84.2%. Explanatory factorial analysis (EFA), multivariate Discriminant Analysis (DA) and Binary Logistic Regression (BLR) served for the statistical analyses.

**Results:**

The percentages of occasional/current regular smokers were 41.1% and 55.7% (*p* = 0.068) among Lithuanian and international male students, and 22.7% and 22.9% (*p* = 0.776) among Lithuanian and international female students, respectively. The international dental students had a deeper knowledge of the harmfulness/addictiveness of smoking and held more positive attitudes towards smoking cessation among their patients than did the native Lithuanian dental students.

**Conclusions:**

The findings of the study underscored the need to properly incorporate tobacco cessation training into the curriculum of dental education. However, consideration of the cultural background of dental students in building up their capacity and competence for intervening against smoking is essential.

## Background

Dental professionals as members of a multi-professional health care team are uniquely positioned to contribute efforts both to prevent smoking and to promote its cessation [1]. Numerous studies have shown that even brief and simple advice from health professionals can substantially lower smoking rates and that prevention and cessation counselling in the dental setting is both relevant and effective [1–3].

Training health professionals to provide smoking cessation counselling increases success rates [4]. Individual factors, which can include the dentists’ own smoking history as well as attitude and cultural background, can then shape individual differences in their patients’ responsiveness to smoking cessation counselling [5, 6]. Dental education can play an important role in building up the courage and competence of dental professionals to intervene against smoking [7], but this would require dental professionals to adopt a positive attitude towards smoking prevention and cessation, systematic education about the health risks linked to it, the dangers of addiction to it, and the provision of treatments for it [8].

Smoking behaviour as well as attitudes towards and social norms regarding tobacco use vary greatly across countries and different cultures. Dental students’ tobacco use in certain countries differs and varies widely [9]. Dental students’ attitudes towards anti-smoking programmes vary considerably, and such formal training in dental education is often lacking [9, 10]. Though more than 90% of both German and UK dental students believe that dentists should advise their smoking patients to quit, only 25% of German and 33% of UK students feel competent enough to counsel them to do so (such competence increased with years of study) [11].

Smoking is common among dental students in the Baltic countries [8, 12]. In Lithuania, 16.5% of adolescents reported being current smokers [13], and smoking among adults has traditionally been high (42% among men, 19% among women in 2010) [14].

Immigrants from countries with a lower smoking prevalence tend to adopt unhealthy smoking-related behaviours [15, 16]. We do not know what impact dental education has on students’ attitudes towards smoking and what role their cultural background plays in smoking cessation. Since provision of parallel dental education in English for international students has become very common especially in the former “Eastern Europe” the general aim of our study was to compare native Lithuanian and international dental students’ smoking habits, knowledge about the harmfulness of smoking and attitudes towards smoking cessation.

## Methods

We carried out a cross-sectional survey of smoking and its cessation among all dental students in the Lithuanian University of Health Sciences (Kaunas, Lithuania). The Bioethics Center of Lithuanian University of Health Sciences granted their approval of the ethics of the study protocol (reference: BEC-OF-445). The Lithuanian University of Health Sciences (LUHS) has a five-year curriculum in dentistry and in the 1990s began offering dental education in English for international students using a parallel and identical curriculum. The LUHS dental curriculum adheres to the European undergraduate profile and competencies [17], but features no courses dedicated to smoking prevention or cessation.

### Subjects

In March–June 2012, dental researchers (JN, JV) asked all (*n* = 722) dental students (both Lithuanian and international) in each year of their dental education to complete an anonymous self-administered written questionnaire during a compulsory practical class or seminar; participation was completely voluntary and by answering the student consented to the study. After completing the questionnaires, the students immediately returned them to the instructor. A total of 606 students and returned their completed questionnaires (response rate: 84.2%; range among the year of study: 73.5%–90.1%). Altogether 511 (84.3%) Lithuanian and 95 (15.7%) international students participated (response rates: 85.0% and 81.7%, respectively). The proportion of women among the international students was lower than among the native students (35.8% and 79.1%, respectively; *p* < 0.001). The international students were older than the Lithuanian students (percentages of those age 23 (median of total sample) and older were 64.2% and 28.0%, respectively; *p* < 0.001). Because of the significant difference in distribution between these groups, data in the analyses were weighted to adjust for the distribution of respondents by age and gender in the total sample.

### The questionnaire

The questionnaire was based on previous research among dental students [8, 18]. The researchers translated it from English to Lithuanian, and a native English speaker back translated it in an effort to ensure the accuracy of the Lithuanian version.

The first part of the questionnaire used yes/no answers to gather information about the student’s current smoking status (“no”; “yes, daily”; “yes, occasionally”) and smoking history (“smoked at least 100 times during my lifetime”, “never smoked regularly”).

The second part of the questionnaire assessed the respondent’s knowledge of the health risks of tobacco smoking and its addictiveness. A three-item “Harmfulness” scale assessed students’ knowledge of the health risks of smoking (general morbidity, oral health hazards) and of environmental tobacco smoke. The questionnaire enquired about the students’ perceived knowledge of the addictiveness of smoking with a four-item “Addictiveness” scale for measuring physical, psychological, social and habitual (behavioural) dependence. Both scales provided five answer alternatives (“not at all”, “little”, “moderately”, “very” and “extremely”) for the questions.

The third part of the questionnaire enquired about the student’s attitudes towards smoking prevention and cessation as well as their perceptions of the dentist’s role in these. An eight-item “Attitudes” scale included statements such as “Smoking prevention should be part of health care personnel education” or “It is the dentist’s responsibility and duty to try to encourage his or her patients to quit smoking”, using five-point Likert items ranging from “fully disagree” to “fully agree”.

The “Willingness” scale from the last part of the questionnaire served to measure the students’ opinion of their willingness to help their future patients to stop smoking. The scale included five statements, and the students were asked how much (“not at all”, “little”, “moderately”, “very” or “extremely”) they agree with each of these statements. Table 2 presents the full list of the items of the scales. Furthermore, all respondents were asked to indicate their gender, age group and current year of study.

### Statistical analyses

To simplifier and understand better the interrelations among the full set of measures and to confirm the inherent structure of the scales, we performed an explanatory factorial analysis (EFA). Using the SPSS “Factor Analysis” procedure, we conducted a Principal Component Factor analysis that included a Varimax rotation as necessary. The Kaiser-Meyer-Olkin (KMO) measure of sampling adequacy and Bartlett’s test of sphericity (KMO > 0.5 and *p* < 0.001 for an adequate sample) served to evaluate the appropriateness of the factor models. We then extracted the factors based on the breaking point of their successive eigenvalues (> 1), item factor loadings (> 0.4) and interpretability. Goodness of fit was assessed with the percentage of explained variance. Next, we calculated the factor scores for each respondent and saved them for subsequent EFAs. Higher positive values for the factor scores corresponded to more thorough knowledge or a more positive attitude. In addition, Cronbach’s α served as a measure of the internal consistency of the scales; the measures ranged from 0.68 for the “Addictiveness” scale to 0.79 for the “Willingness” scale and can be considered appropriate.

The next step in the statistical analysis served to evaluate similarities/differences in knowledge and attitude profiles between the groups of Lithuanian and international dental students. For this reason, we conducted a multivariate Discriminant Analysis (DA) of the overall data, adjusting for gender, age, year of study and current smoking status. We dichotomised the adjusting variables as follows: the first age group included students up to 22 years of age, and the second age group included students 23 years of age and older, year of study was dichotomised into ‘pre-clinical’ (1st and 2nd years) vs. ‘clinical’ (3rd, 4th and 5th years) phases, and current smoking was dichotomised into ‘smokers’ (daily/occasionally) and ‘non-smokers’. We also calculated standardised Canonical Discriminant Function Coefficients (CDFC) and Structure Coefficients (correlations between discriminating variables and standardised canonical discriminant function). The coefficient values indicated the weight of the corresponding variables (knowledge and attitude factors) in discriminating between students groups. We then compared the results of the DA to the results of the multivariate Binary Logistic Regression (BLR), in which the student group (international vs. Lithuanian) served as a dependent variable. In this analysis, we calculated the Odds Ratios (OR) and their 95% Confidence Intervals (95% CI) of the factors in discriminating international vs. Lithuanian dental students. The significance level was set at *p* ≤ 0.05. We used SPSS (version 20.0; SPSS Inc., Chicago, IL, 2010) to carry out the data analysis.

## Results

Table 1 shows the results of the survey on current and previous smoking among Lithuanian and international dental students. Current smoking was more prevalent among international students than among Lithuanian students, although the difference in smoking rates was non-significant. The percentages of occasional/current regular smokers were 41.1% and 55.7% (*p* = 0.068) among the Lithuanian and international male students, and 22.7% and 22.9% (*p* = 0.776) among the Lithuanian and international female students, respectively.

**Table 1:** Current and previous smoking among the Lithuanian and international dental students by gender

	Male^a^				Female^a^				Total^b^			
	Lithuanian *N* = 107		International *N* = 60		Lithuanian *N* = 404		International *N* = 34		Lithuanian *N* = 511		International *N* = 94	
	n	(%)	n	(%)	n	(%)	n	(%)	n	(%)	n	(%)
*Current smoking:*												
Non-smoker	63	(58.9)	27	(44.2)	313	(77.3)	27	(77.1)	370	(72.3)	65	(68.4)
Occasional smoker	24	(22.4)	17	(27.9)	72	(17.8)	5	(14.3)	97	(19.0)	16	(16.8)
Regular smoker	20	(18.7)	17	(27.9)	20	(4.9)	3	(8.6)	44	(8.7)	14	(14.7)
Occasional/regular smoker	44	(41.1)	34	(55.7)	92	(22.7)	8	(22.9)	142	(27.7)	30	(31.6)
*Previous smoking:*												
Have smoked at least 100 times	58	(54.2)	35	(57.4)	128	(31.7)	4	(11.8)*	193	(37.8)	23	(24.5)*
Have ever smoked regularly at least one year	31	(29.2)	29	(47.5)*	62	(15.3)	5	(14.7)	98	(19.2)	22	(23.2)
Have tried to quit smoking at least once	26	(59.1)	26	(74.3)	43	(48.9)	2	(28.6)	73	(52.9)	15	(50.0)

^a^Data weighted by age

^b^Data weighted by age and gender

*p < 0.05 comparing to Lithuanian dental students

Having smoked at least 100 times was more common among the Lithuanian female students than among their international peers (31.7% vs. 11.8%; *p* = 0.015), while having smoked regularly for at least one year was more common among the international than among the Lithuanian male students (47.5% vs. 29.2%; *p* = 0.018). Rates of attempts to quit smoking between the Lithuanian and international students showed no significant differences.

### Factor analysis of scales for knowledge and attitudes

Table 2 presents the results of the EFA of the total study sample weighted by age and gender. The appropriateness of the factor models evaluated with the KMO measure of sampling adequacy and Bartlett’s test of sphericity can be considered meritorious. The “Harmfulness”, “Addictiveness” and “Willingness” scales had homogenous structures, so we simplified their dimensions down to a single factor, which accounted for 66.9%, 51.8% and 54.2% of the total item variation in the corresponding scales. Factor analysis of the “Attitudes” scale revealed a two-factor solution, which accounted for 50.3% of the total variance. The estimated loadings indicated that the first component combined four items. We found the maximal loading (0.84) for the item “Smoking prevention should be part of health care personnel education”. The remaining three items also had great loadings of the first component. We interpreted this group of items as a factor influencing “dentists’ knowledge and skills”. The second factor combined four items clearly distinguished by great loadings of the second component. Agreement with these statements indicated an attitude of greater striving to help their patients to quit smoking. We identified this component as a factor for “helping patients”.

**Table 2:** Results of the internal consistency and explanatory factor analysis^a^ of the scale

Scales, items^b^, and measures	Factor 1	Factor 2
*Harmfulness (of smoking)*		
*Cronbach’s α = 0.73 KMO measure: 0.67; Bartlett’s test: p < 0.001*		
How harmful is smoking to oral health?	0.86	
How harmful is smoking to health?	0.81	
How harmful is environmental cigarette smoke to health?	0.77	
*Total variance explained by a Factor*	66.9%	
*Addiction (to smoking)*		
*Cronbach’s α = 0.68 KMO measure:0.71; Bartlett’s test: p < 0.001*		
Habitually addictive	0.77	
Psychologically addictive	0.73	
Physically addictive	0.70	
Socially addictive	0.67	
*Total variance explained by a Factor*	51.8%	
*Willingness (to patients’ smoking cessation)*		
*Cronbach’s α = 0.79 KMO measure: 0.75; Bartlett’s test: p < 0.001*		
Are you willing to use anti-tobacco programs in your own practice? (e.g. flyers, advisement, … etc.)	0.77	
Are you willing to show your patients the damage smoking can cause upon general health?	0.75	
Are you willing to advise patients to stop smoking?	0.75	
Are you willing to cooperate actively in anti-tobacco programs on community level?	0.74	
Are you willing to show your patients the damage that smoking can cause upon oral health?	0.68	
*Total variance explained by a Factor*	54.2%	
*Attitude (towards patients’ smoking cessation)*		
*Cronbach’s α = 0.69 KMO measure: 0.70; Bartlett’s test: p < 0.001*		
*Items related to Factor 1:*		
Smoking prevention should be part of health care personnel’s education	0.84	0.15
Health care personnel should get special training to help patients willing to quit smoking	0.83	0.14
It is a dentist’s responsibility and duty to try to get patients to quit smoking	0.62	0.21
My knowledge and skills are sufficient to guide patients who want to quit smoking	0.56	−0.06
*Items related to Factor 2:*		
The main obstacle for quitting smoking is insufficient information about cessation methods	0.08	0.79
The main obstacle for quitting smoking for patients is insufficient information about risks of smoking	0.06	0.71
Patients need mainly practical tips on how to quit smoking	0.03	0.66
Support from a health professional is needed to quit smoking	0.18	0.50
*Total variance explained by a Factor*	26.5%	23.8%

^a^Extraction method: Principal Component Analysis; Rotation method: Varimax

^b^Items were sorted by descending values of loadings

The factors correlated positively, with the strongest correlations occurring between the factors “Harmfulness” and “Addictiveness” (*r* = 0.376; *p* < 0.001), “Harmfulness” and “Willingness” (*r* = 0.345; *p* < 0.001), and “Willingness” and “Dentist’s knowledge and skills” (*r* = 0.255; *p* < 0.001).

### Differences in Lithuanian and international students’ knowledge of and attitudes toward smoking

The means of factor scores of the knowledge and attitude factors, with the exception of the “Help patients” factor, were significantly higher among international students than among Lithuanian students (Table 3). These findings shows that international students were more likely than Lithuanian students to report higher scores for sharing their opinions about the harmfulness of smoking and the addictiveness of smoking, more willing to discourage smoking among their patients, and more likely to agree with the need to improve dentists’ knowledge and skills in counselling their patients against smoking. The highest difference (0.60; *p* < 0.001) was in the factor “Dentists’ knowledge and skills”.

**Table 3:** Weights of knowledge and attitude factors in discriminating Lithuanian and international dental students towards smoking^a^

Discriminating variables	Group means		Wilks’ Lambda	F	p	Standardized canonical discriminant function coefficients	Structure coefficients
	Lithuanian	International					
*Knowledge and attitude factors:*							
Harmfulness (of smoking)	−0.03	0.18	0.994	3.74	0.054	−0.015	0.250
Addiction (to smoking)	−0.06	0.34	0.978	13.58	<0.001	0.520	0.476
Willingness (to patients’ smoking cessation)	−0.04	0.18	0.994	3.86	0.050	−0.031	0.254
“Dentist’s knowledge and skills”	−0.09	0.51	0.951	31.17	<0.001	0.733	0.721
“Help patients”	0.01	−0.02	1.000	0.05	0.824	−0.116	−0.029
*Adjusting variables:*							
Age (% of 23+ year olds)	33.7	33.7	1.000	0.00	0.988	0.214	0.002
Gender (% of female)	72.4	72.4	1.000	0.00	0.991	−0.176	0.001
Study phase (% of clinical students)	58.9	42.6	0.985	9.01	0.003	−0.585	−0.388
Smoking (% of smokers)	27.7	31.6	0.999	0.61	0.612	0.050	0.101
Functions at Group Centroids	−0.14	0.73		59.87	<0.001		

^a^Data weighted by age and gender

To evaluate the factors’ weights in discriminating between the Lithuanian and international students’ knowledge of and attitudes towards smoking, we conducted a multivariate DA (Table 3). The factor of students’ attitudes towards “Dentists’ knowledge and skills” had the maximal values of the standardised CDFC and structure coefficient, so this factor proved to be the most significant variable. The factor of students’ knowledge about addiction to smoking was also an important variable. The factors “Harmfulness” and “Willingness” were of borderline significance. The positive sign of assessed coefficients indicated that international students had higher values for knowledge and attitude factors. The model also included adjusting variable for age, gender, study phase and current smoking status. Among these variables, “Study phase” had a negative significant coefficient for the canonical discriminant function, because the clinical phase of studies was more common among Lithuanian dental students. Students’ current smoking status had minimal weight in the value of the canonical discriminant function.

Table 4 presents the results of the multivariate BLR analysis, in which the selected group of students (Lithuanian vs. international) served as a dependent variable. This method once again allowed to examine the discriminating values of knowledge and attitudes factors between Lithuanian and international students, thereby confirming the results of the multivariate DA. As is evident, the factor of students’ attitudes towards “Dentists’ knowledge and skills” and the factor of students’ knowledge of addiction to smoking proved to be significant predictors of the students group. Incrementing these factors increases the odds of being international student in discriminating students’ groups. The role of adjusting variables was equivalent to those of the DA model.

**Table 4:** Results of multivariate logistic regression analysis^a^ of knowledge and attitude factors in discriminating international vs Lithuanian dental students

Discriminating variables	B	p	OR	95% CI for OR	
				Lower	Upper
*Knowledge and attitude factors (continuous variables):*					
Harmfulness (of smoking)	−0.02	0.901	0.98	0.74	1.31
Addiction (to smoking)	0.47	0.001	1.60	1.20	2.13
Willingness (to patients’ smoking cessation)	−0.04	0.768	0.96	0.75	1.24
“Dentist’s knowledge and skills”	0.76	<0.001	2.14	1.59	2.88
“Help patients”	−0.12	0.331	0.89	0.70	1.13
*Adjusting (categorical) variables* ^*b*^ *:*					
Age (≤ 22-year olds)	0.41	0.176	1.50	0.83	2.70
Gender (male)	−0.33	0.245	0.72	0.41	1.26
Study phase (pre-clinical)	−1.04	<0.001	0.35	0.20	0.62
Smoking (non-smokers)	0.01	0.972	1.01	0.59	1.73

*OR* Odds Ratio of corresponding variable in discriminating international vs Lithuanian students. *CI* Confidence Interval

^a^Data weighted by age and gender

^b^In brackets: Reference Category

## Discussion

The primary aim of this study was to determine and to compare native Lithuanian and international dental students’ smoking habits, their knowledge of the harmfulness of smoking, and their attitudes towards smoking cessation. The results revealed no significant differences in the prevalence of current smoking habits between the native and the international dental students, although the international dental students had deeper knowledge of the harmfulness/addictiveness of smoking and held more positive attitudes towards smoking cessation among their patients than did the native Lithuanian dental students.

The Global Health Professions Student Surveys (GHPSS) conducted among medical and dental students in 47 countries showed that, on average, over 20% of the students currently smoked cigarettes, with men smoking at higher rates than women [9, 19]. A majority (> 60–70%) of the students reported being exposed to second-hand smoke in public places. In a recent study from Latvia, about one-fourth of dental students (24%) were daily or occasional smokers, and half of the male students (46%) had smoked at least 100 times in their lifetime [8]. Among our Lithuanian and international dental students, 28% and 32% were daily or occasional smokers, respectively. These findings are a bit higher than the GHPSS average, but in line with the findings from Latvia and Romania [8, 20]. These findings also concur with a report on smoking among Lithuanian adolescents in which 17% of 13- to 15-year-olds reported being current smokers, with a significantly higher percentage among boys than among girls (20.8% vs. 11.9%) [13].

### The role of dental education

Smoking is the most important preventable cause of a number of general diseases and is a serious oral health problem [21]. As health professionals working closest to the mouth and having unmatched opportunities to identify smokers, dentists are well positioned to propose to and advise their patients to undergo tobacco dependence treatment. Numerous studies have shown that advice from health professionals can substantially reduce smoking [1–3]. However, medical and dental schools often provide only basic knowledge-based tobacco education, if any, in their curricula [22, 23]. Tobacco dependence education (TDE), for instance, is not a component of the curriculum in all US and Canadian dental schools, and such curricula seldom incorporate effective behaviour-based components that effect long-term change [22, 23]. Faculty members are confident in teaching tobacco-related pathology, but lack the interest and skills needed to integrate TDE into patient care [22] or have limited formal training in TDE themselves [24]. Specific TDE and tobacco cessation training is not part of the dental curricula at LUHS.

The GHPSS data on 28,420 health profession students (medical, dental, nursing, and pharmacy) showed that the dental students reported clearly lower competence to intervene in patients’ tobacco use than did the medical students [25]. This lack of training among oral health care students is reflected among practising dental professionals, who continue to report low rates of tobacco interventions [23]. Dental health education should place more emphasis on dental health professionals offering smoking counselling and cessation to their smoking patients [26]. Ramseier et al. [27] has suggested including the following in the dental curriculum: (1) the biological effects of tobacco use, (2) the history of tobacco culture and psychosocial aspects of tobacco use, (3) the prevention and treatment of tobacco use and dependence, and (4) the development of clinical skills for tobacco use prevention and cessation [27]. Studies have shown that engaging dental students in smoking cessation with motivational interviewing and, for example, a video feedback-based cessation counselling programme is suitable for undergraduate dental education [7, 28]. Thus the findings of our and other studies highlight the need for enhanced measures to incorporate tobacco cessation training as a formal component of dental education utilizing a multiprofessional approach.

### Attitudes and culture

The dental students generally held positive attitudes towards smoking prevention and cessation [8, 29]. In Latvia, for instance, 84% of dental students indicated that these should be part of the education provided for health care personnel [8]. Final-year dental students showed well established, favourable oral hygiene attitudes and behaviours, with evidence suggesting that their knowledge developed whilst in dental school [29]. As in other studies, the participants in our study also indicated that smoking prevention should be part of the education of health care personnel [8, 9].

Our students were generally aware of the harmfulness of smoking to health and oral health, and showed openness to smoking cessation; however, they perceived their knowledge and skills as inadequate to advise their patients to quit smoking. In addition, dental students tended to have a poor understanding of the effectiveness of smoking cessation counselling in the dental setting [10]. In our study, the international students were more willing than the native Lithuanian dental students to counsel their patients to stop smoking. The greatest difference occurred in the items “Health personnel should receive special training to help their patients to quit smoking” and “It is a dentist’s responsibility and duty to counsel his or her patients to quit smoking”. In addition, the international students showed higher awareness of the harmfulness and addictiveness of smoking, as well as confidence in their skills in regards to the item “My knowledge and skills are adequate to guide patients who want to quit smoking”. The opposite was seen in the “Help patients” item, where more Lithuanian than international students indicated that “The main obstacle to quitting smoking is insufficient information about cessation methods”, “The main obstacle to quitting smoking among patients is insufficient information about the risks of smoking”, and “Support from a health professional is needed to quit smoking”.

The dental students’ smoking experience and current smoking as well as social dependence associated with their attitudes [20, 30]. The findings that the international students were more aware of the harmfulness and addictiveness of smoking, and were more willing to help patients to stop smoking than were the native students could stem from cultural differences and background. A considerable number of the international students were from the Eastern Mediterranean, where smoking is socially more accepted among men than among women [9, 30].

### Strengths and limitations

To our knowledge, this is the first study to compare national and international students in the same curriculum. The study used pretested questionnaires among dental and medical students and professionals in several countries [8, 18, 31] and covered all study years with a response rate of 84.2%, which can be considered high. This study is a step forward towards incorporating tobacco cessation training into dental curricula. However, this survey has some inherent limitations. Firstly, we collected the data with a self-reported questionnaire and, as with all questionnaires, the possibility of both intentional and unintentional misreporting can compromise the validity and reliability of the findings. To cope with such a potential bias, we took special measures to ensure the anonymity of the respondents and to keep the study anonymous. Secondly, the international students’ multinational backgrounds (almost half of the students were from Eastern Mediterranean region, about one third from Eastern and Western European countries, one fifth from Asia regions) limited assessments of the impact of national characteristics on the research problem.

## Conclusions

The international dental students had deeper knowledge of the harmfulness of smoking and held more positive attitudes towards smoking cessation among their patients than did the native Lithuanian dental students. The findings of our study underscored the need to incorporate tobacco cessation training as a formal component into the dental education curriculum. However, it is essential to take into consideration the dental students’ cultural backgrounds in developing their capacity and competence to intervene against smoking.

## Abbreviations

BLR: Binary logistic regression
CDFC: Canonical discriminant function coefficients
CI: Confidence interval
DA: Discriminant analysis
EFA: Explanatory factorial analysis
GHPSS: Global health professions student survey
LUHS: Lithuanian University of Health Sciences
OR: Odds ratio
